# Information System and Geographic Information System Tools in the Data Analyses of the Control Program for Visceral Leishmaniases from 2006 to 2010 in the Sanitary District of Venda Nova, Belo Horizonte, Minas Gerais, Brazil

**DOI:** 10.1155/2012/254361

**Published:** 2012-02-07

**Authors:** Lara Saraiva, Camila Gonçalves Leite, Luiz Otávio Alves de Carvalho, José Dilermando Andrade Filho, Fernanda Carvalho de Menezes, Vanessa de Oliveira Pires Fiúza

**Affiliations:** ^1^Centro de Referência Nacional e Internacional para Flebotomíneos, Centro de Pesquisas René Rachou, 30190-002 Belo Horizonte, MG, Brazil; ^2^Prefeitura Municipal de Belo Horizonte, Secretaria Municipal de Saúde, Gerência de Controle de Zoonoses do Distrito Sanitário Venda Nova, 31520-000 Belo Horizonte, MG, Brazil

## Abstract

The aim of this paper is to report a brief history of control actions for Visceral Leishmaniasis (VL) from 2006 to 2010 in the Sanitary District (DS) of Venda Nova, Belo Horizonte, Minas Gerais, Brazil, focusing on the use of information systems and Geographic Information System (GIS) tools. The analyses showed that the use of an automated database allied with geoprocessing tools may favor control measures of VL, especially with regard to the evaluation of control actions carried out. Descriptive analyses of control measures allowed to evaluating that the information system and GIS tools promoted greater efficiency in making decisions and planning activities. These analyses also pointed to the necessity of new approaches to the control of VL in large urban centers.

## 1. Introduction

According to the classification of World Health Organization [1], leishmaniases are neglected diseases of great epidemiological importance, which require effective control measures. They present potential to epidemic outbreaks because of their transmission by vector insects [2].

These diseases present a wide variation in their geographical areas of occurrence, with a focal distribution. Leishmaniases affect more than 100 countries worldwide, with tropical or subtropical climate occurring zoonotic and anthroponotic cycles [2–4]. VL, also referred as kalazar, attacks the internal organs being the most severe pathology. VL is a zoonotic disease spread worldwide and the highest concentration of cases (90%) occurs in six countries including Bangladesh, India, Nepal, Sudan, Ethiopia, and Brazil [5].

In the Americas, around 90% of human cases of VL are registered in Brazil, where 21 (77.8%) of its 27 federative units have reported autochthonous cases [6]. VL was originally a rural disease in Brazil, however, around 20 years ago, it has been spreading to urban areas of medium and large sizes [7]. 

**Figure 1:**
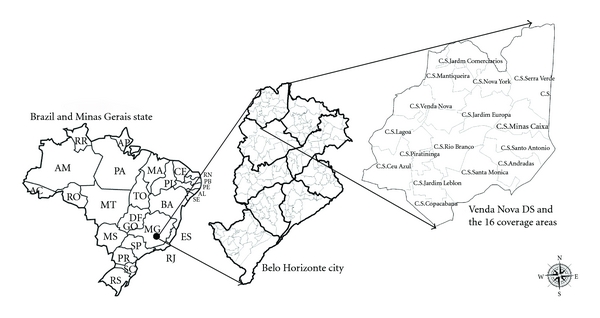
Administrative division of the Municipal District of Belo Horizonte and its location in Minas Gerais and in Brazil and Venda Nova Sanitary District location in the city of Belo Horizonte.

Belo Horizonte (BH) City, the capital of Minas Gerais State, is located between the coordinates 19° 55′ South and 43° 57′ West, and at 858 meters above the sea level. The climate is tropical, average annual temperature is 21°C, and average rainfall is 1.450 mm/year. There are 2.412.937 inhabitants distributed in 331 km^2^ with a population density of 7.209,08 inhabitants per km^2^ [8]. The city is the main pole of services, knowledge, and technology of Minas Gerais. BH city is divided into nine sanitary districts (SD) which have defined geographical area, population, and administrative staff [9]. Each SD is subdivided into coverage areas (CAs), which correspond to the territory attended by a primary public health care unit (Public Health Centers), see Figure 1. Metropolitan region of BH is composed by 33 municipalities that also have geographical proximity (often being adjacent) and interchanging of inhabitants, products, and input with the capital. Autochthonous cases of VL have been registered by the official health service since 1989 in the metropolitan region of BH, Minas Gerais, which have a resident population predominantly urban [10]. 

 The first autochthonous case of LV occurred in 1994, in Belo Horizonte City in an area bordering the municipality of Sabará. The disease has spread gradually in the following years to all regions of BH, presenting a pattern of transmission typically urban (domiciliary and peridomiciliary). VL has become a serious public health problem and a priority program of municipal government of BH City, due to the high incidence and lethality rates and geographic spreading. The Municipal Secretariat of Health of Belo Horizonte (MSH) reported 1,434 cases (partial data) of the disease with 161 deaths (11% of lethality), and VL cases have been reported in all sanitary districts from 1994 to 2010 [9].

Venda Nova SD—one of the nine SDs that comprise administrative division of BH—is the oldest one located in the north region of BH (Figure 1). Considering the period from 2006 to 2010, Venda Nova SD reported 104 cases, with 9 deaths (9% of lethality). The history of the human disease in Venda Nova SD shows that since the occurrence of the first autochthonous case of human VL in 1994, there were a territorial expansion and an increase in the number of cases. This also could be observed for canine infection rates [9].

The geographical expansion and increasing number of VL cases in the municipality have determined the rising demand of the program to control the disease leading to the need for agile and reliable analyses of data generated by performed actions. In this regard, the MSH in partnership with the Data Processing Company of Belo Horizonte (Prodabel) developed and implemented a computerized system to register the activities of the Programme for the Control of Visceral Leishmaniasis—SCZOO LV in 2006. Data have been produced by the control activities of the canine reservoir (blood collection, laboratory processing, collection of serologically positive dogs, and euthanasia of seropositive dogs) and they are inserted into the Information System for Control of Zoonoses—component Visceral Leishmaniasis—subcomponent Canine Enquiry (SCZOO/LV/IC) [11].

The data of chemical control activities are inserted in the subcomponent of Insecticide Operation SCZOO/LV, which started in 2009, as a pilot project, being implemented in all the SDs from 2011. The data consolidation about the chemical measures was previously inserted on form called SD Weekly Leishmaniasis, centralized in the MHS. Both subcomponents of SCZOO/LV perform the automatic georeferencing of the data and generate the files in  .dbf format which may be analyzed through the software Map Info, available in all SDs of Belo Horizonte.

## 2. Materials and Methods

This work aimed to report the experience of control of VL in Venda Nova SD, Belo Horizonte. The data analyzed were obtained from the Information Systems Control of Zoonoses—SCZOO/VL, subcomponent Enquiry Canine and Insecticide Operation, the Brazilian Information System for Reportable Diseases, and technical reports of the Management Control of Zoonoses of the SMH of Belo Horizonte and Management of Zoonoses Control (MZC) of Venda Nova SD.

The study analyzed data of control measures adopted by Venda Nova SD for Visceral Leishmaniasis from 2006 to 2010, considering the following aspects: epidemiological situation, the stratification of risk areas, annual programming of activities to control the canine reservoir and chemical control of vectors, geoprocessing of data, and new control strategy approaches.

### 2.1. Study Area

Venda Nova SD is the oldest region of Belo Horizonte a thirty- year- old region. The origin and formation date back to 1711, when the troops of muleteers carried food and cattle to supply the district called “Curral D'el Rey.” The SD has a population of 263,930 inhabitants [12], an area of 28.30 km^2^, and a population density of 8,670.58 inhabitants per km^2^. The dog population is 36,706 dogs (1 dog : 7 inhabitants), according to the canine census of 2010 [Sanitary District Data, unpublished]. The region has 64,894 human dwellings, 89% are horizontal houses, however, there are prospects for verticalization. Characteristically, the occupation is heterogeneous with a variation between formal areas and peculiar environments such as houses with large gardens and terrain of great extension, places where individuals cultivate vegetables and raise livestock animals, and areas occupied by poverty pockets. The altitude ranges from 751 to 1000 meters above the sea level. The SD is subdivided into 16 coverage areas (AA): Céu Azul, Andradas, Venda Nova, Copacabana, Rio Branco, Santa Monica, Piratininga, Santo Antonio, Serra Verde, Minas Caixa, Jardim Europa, Jardim Leblon, Nova York, Mantiqueira, Minas Caixa, and Jardim dos Comerciários (Figure 1).

### 2.2. Georeferencing

Databases georeferencing was performed using the coordinate system UTM and the datum SAD 69 (South American Datum). Geocoding was accomplished by SCZOO whether base did not have the coordinates of the address case, neighbours coordinates were used. Spatial analyses were carried out using MapInfo 8.5 Professional. These procedures are descriptive and based on overlaying maps and cluster analyses (hot spot) with a radius of 400 meters (average size of a city block in BH) and resolution of 80 meters. The procedures used are performed in the routine control of Management of Zoonosis Control (MZC).

### 2.3. Result Analyses

Files (in  .dbf format) of SCZOO were organized through Excell 97/2003 generating compatible files with the software MapInfo and Prism. Excell 97/2003 was used to the descriptive statistics too. Graph Pad Prisma 4.0 was used for statistical analyses.

## 3. Results and Discussion

Evaluation of the annuals control measures for Visceral Leishmaniases in Venda Nova SD is reported here.

### 3.1. 2006

**Figure 2:**
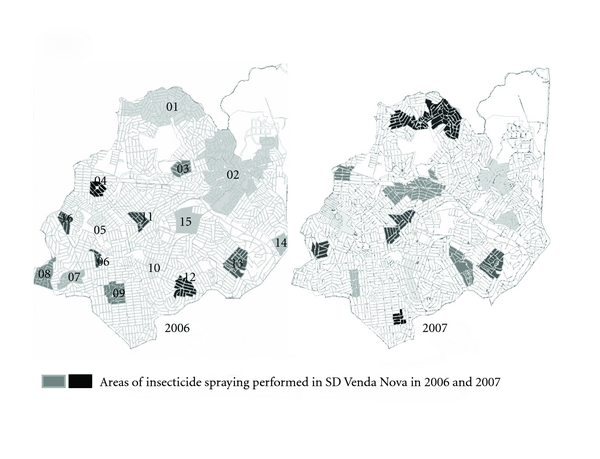
Example of the geographical pattern of areas scheduled for insecticide spraying in SD Venda Nova in the years from 2006 to 2010. The areas shown were planned for the years 2006 and 2007.

 Venda Nova SD carries out actions of control of VL in the priority areas (according the occurrence of human cases), but spraying of insecticides in buildings was also done where serologically positive dogs had been collected for euthanasia in 2006. A total of 31,639 buildings have been programmed for spraying, however, only 18,076 have been sprayed indicating the low coverage of insecticide spraying and the disparity between the number of buildings scheduled and those effectively sprayed. Sprayed areas have a dispersed profile without coverage of contiguous areas (Figure 2). In addition, the single insecticide spraying in buildings with serologically positive dogs presents low effectiveness in control. Whereas the insects can be only dispersed because places surrounding the sprayed building remain the same environment. A total of 12,072 serological tests for detection of *Leishmania* sp. were performed in serosurvey of dogs, with 11.57% positivity rate. Out of these positive dogs, 87% were euthanatized by the MCZ (Figures 2 and 7).

SCZOO was implemented in the SD in this year. This software automatically georeference all dogs examined and report their situation. The analyses of control measures were already available through the data of this database after 2006.

### 3.2. 2007

**Figure 3:**
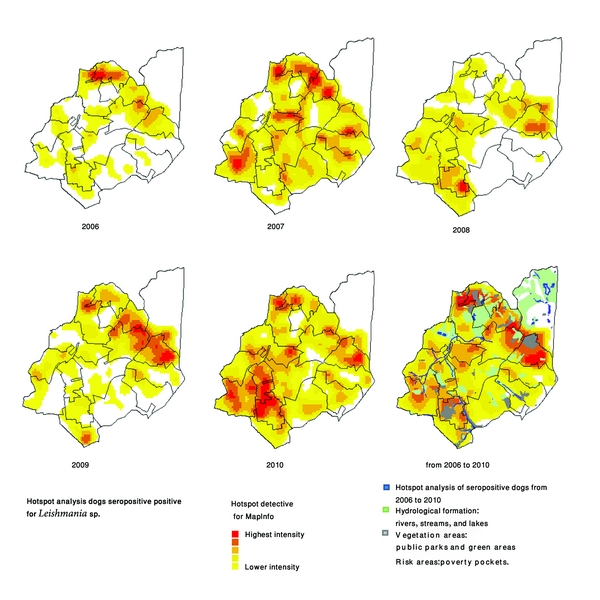
Dogs seropositive for *Leishmania* sp. in serosurveys carried out in SD Venda Nova from 2006 to 2010, the results shown georeferenced positive samples and clusters analysis for each years.

Program to 2007 remained the same pattern carried out for 2006, 16,716 buildings were planned to be sprayed and only 10,226 were sprayed, and buildings maintained the same pattern of geographic distribution. However, considering actions to control canine reservoir, there was an increase in number of examinations performed and the euthanasia rate remained high. A total of 24,782 examinations were done in the serosurvey with positivity rate of 11.57% and over 81% of dogs were euthanized by MCZ (Figures 3 and 7). 

This higher number of tests carried out is due to the possibility of more robust analyses, with the software SCZOO. The concern about controlling the canine reservoir led to an increase in processing capacity of samples by the laboratory of the SMH.

### 3.3. 2008

**Figure 4:**
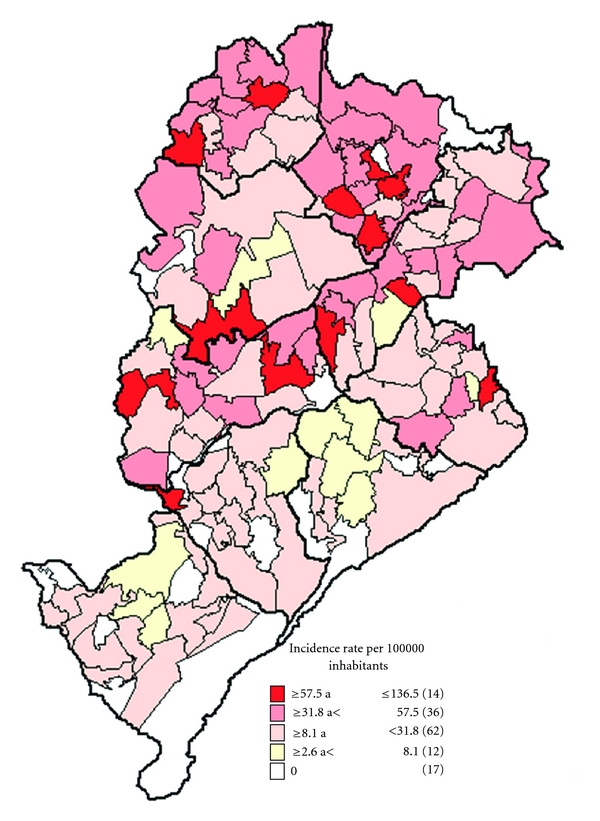
Areas of risk for the transmission of Visceral Leishmaniases in Belo Horizonte City, considering the incidence rates from 2003 to 2007.

The Ministry of Health drew up the Municipal Plan for Intensification of the Surveillance and Control of Visceral Leishmaniases due to the high number of cases of visceral leishmaniases in 2008 (Figure 8). This plan gives priority to efforts to control VL in each covered area (CA). SMH carried out a stratification analysis of risk areas for VL in Belo Horizonte following the warning of Ministry of Health (Figure 4). This analysis was used to recast control measures of VL in Venda Nova SD in 2009. The stratification of incidence rates from 2003 to 2007 show clearly the worrying situation of the disease in Venda Nova SD.

Agents controlling endemics diseases, who work specifically in the control of VL, have been displaced to perform actions to control dengue transmissions due to an increase in number of cases in 2008 despite the Plan for Intensification. Dengue is a disease of epidemic patterns and great political appeal (Figure 9). This fact hindered the measures to control the VL.

Taking into account the 30,000 buildings planned for insecticide spraying, only 4,848 buildings have been sprayed. Insecticide spraying kept the profile geographically dispersed as occurred in 2006 and 2007. A total of 25,087 examinations for *Leishmania* sp. were conducted in a serosurvey of dogs, with a positivity rate of 8.39 and 87.56% of these dogs were euthanized by MZC. Venda Nova SD has had the improvements implemented by the plan of intensification only in 2009.

### 3.4. 2009 and 2010

The team of agents to control the VL received an increase of 24 employees in 2009 as a consequence of intensifying the plan implemented in 2008. The program of control measures has considered operating factors such as human and material resources, dislocation of employees from the health centers to work field, among others in 2009 and 2010, through the analyses of data from previous years. Furthermore, the programs of VL control for 2009 and 2010 were based on the risk stratification (Figure 4) and on Venda Nova SD-specific data such as annual history of VL human cases and annual history rates of seropositive dogs. Analyzed variables were ranked, thus higher weights were attributed to the incidence rates of VL human cases and positivity of dogs exams in the previous year (2008 or 2009).

A serosurvey was carried out in 100% of the domiciled dog population of 11 CA in 2009 and the serosurvey was done in 16 CA (all area of Venda Nova DS) in 2010 considering the logical to work the areas more completely. Discrepancy between the number of buildings programmed for insecticide spraying and the number of buildings effectively sprayed was smaller than the previous years. Despite the possibility of analyzing, the areas of spraying remained the same profile to previous years, covering small and sparingly geographical areas.

A total of 8,000 buildings were scheduled for insecticide spraying in 2009, the procedure was performed in 8,530 buildings. Considering the actions aimed at controlling the canine reservoir, 21,351 tests were carried out in serosurvey, with positivity rates of 7.96% and 89.82% of seropositive dogs were euthanized. A total of 15,000 buildings were programmed for insecticide spraying in 2010 and the procedure was performed in 10,112 properties. The recruitment of employees for actions to control dengue impaired to achieve the goal. BH city has undergone great dengue epidemics in 2010 (Figure 9). Regarding the actions aimed to control the canine reservoir, 30,460 tests were carried out in serological screening, with a positivity rate of 9.36% and 85.70% of the seropositive dogs were euthanized.

### 3.5. Changes to the Control Programming in 2011

**Figure 5:**
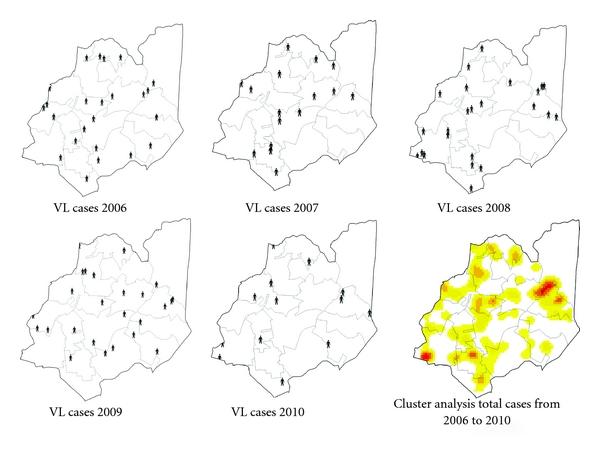
Human cases of Visceral Leishmaniases SD Venda Nova from 2006 to 2010, cluster analysis considering all cases from 2006 to 2010.

The analyses of the incidence rates of VL human cases, the lethality of the disease, and its geographic spread in Venda Nova SD revealed the necessity to employ new approaches in control of VL (Figures 2 and 5). Data of control measures referring to canine reservoir showed that Venda Nova SD performs a significant number of serological tests in its canine population (2006: 32.0% of the population was tested, 2007: 69.5%, 2008: 68.3%, 2009: 58.0,%, 2010: 82.9%).

A large percentage (always higher than 80%) of the serologically positive dogs was euthanized in the years analyzed (Figure 7). Despite these actions, the data point out that the rate of canine seropositive remains high with significant increase in 2010 (Figure 10). Seropositive dogs are dispersed over all areas of the Venda Nova SD, which is evident considering the increase in number of examinations performed and better spatial coverage of the SD, from 2006 to 2010 (Figure 3).

**Figure 6:**
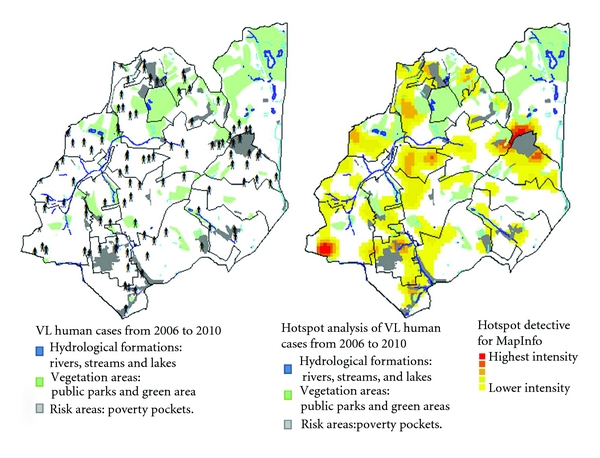
Map of VL human cases from 2006 to 2010 overlaying the maps of Vegetation (green areas and parks), Hydrographic (rivers, streams and lakes) and poor areas and social risk.

Serological canine positivity rate and VL human cases have low linear correlation (r2-0028), in the other words, a human case of VL is a complex event that cannot be explained only by the presence of infected dogs in an area. Overlaying the map of VL, human cases with maps of factors traditionally related to VL as vegetation, hydrography, and areas of poverty pockets. It is not possible to identify a strong spatial correlation between them. Cases of Visceral Leishmaniases are spread in every coverage areas of Venda Nova SD and it is possible to observe areas of concentration of cases both in risk areas and outside them. Considering this data, we may infer that the VL presents new profile of occurrence in urban areas (Figure 6).

Considering these data, we can presume that only the actions for elimination of the vertebrate reservoir and insecticide spraying in areas with human cases and seropositive dogs are not sufficient for an effective control of this endemic disease.

These analyses have only been feasible through the use of SCZOO and by training in MapInfo for technical professionals of SD Venda Nova. These analyses made possible the changes of programmed actions for 2011. Areas were ranked according to geographical history of human cases and seropositive dogs. Canine serosurvey was programmed for 100% of the domiciled dog population and the insecticide spraying was programmed in broader and contiguous geographic areas.

Moreover, the DS prepared a project of Environmental Management and implemented with the Center for Zoonosis Control a projects of castration of dogs domiciled, both project following the criterion of priority areas.

## 4. Final Considerations 

The process of territorial expansion and increasing the number of VL human cases in Belo Horizonte City ordered the municipal management to development and gradual implementation of an Operating Plan to reorganize the actions of disease control in the nine SD of the city [9].

**Figure 7:**
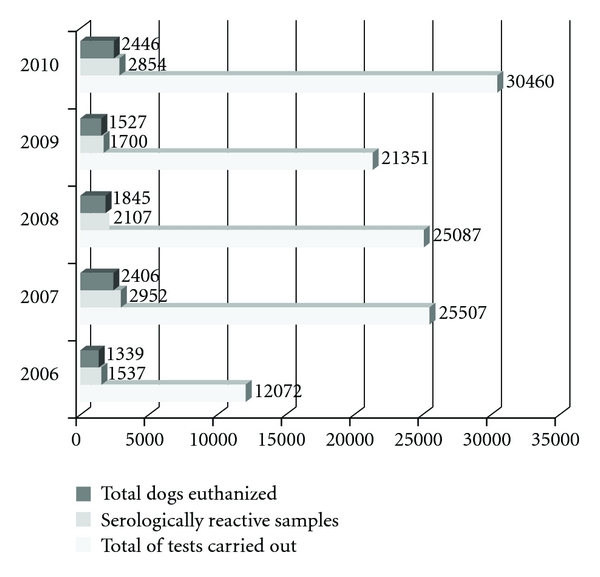
Total number of canine serological tests for *Leishmania* sp performed, total of positive samples, and total of dogs euthanized in SD Venda Nova from 2006 to 2010.

**Figure 8:**
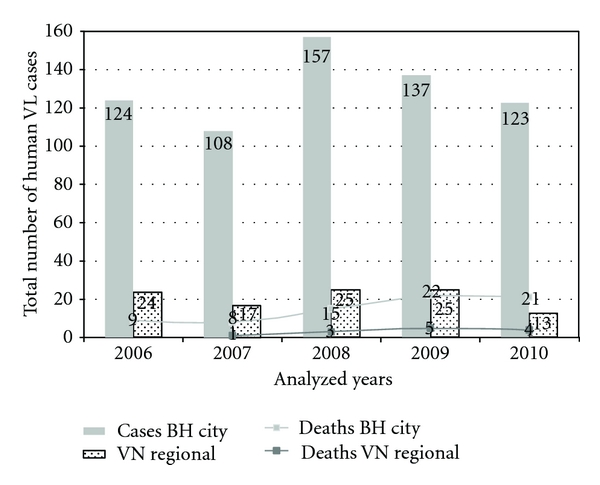
Cases and deaths of Visceral Leishmaniases in Belo Horizonte City and in the Venda Nova Sanitary District from 2006 to 2010.

**Figure 9:**
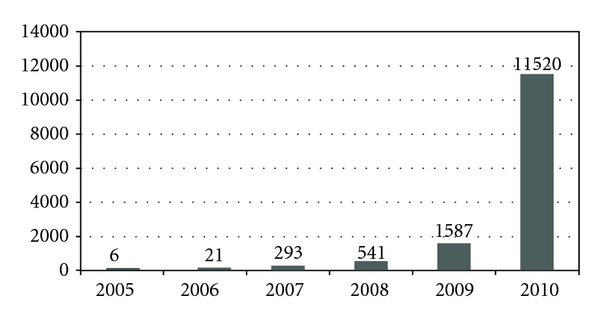
Cases of dengue fever in SD Venda Nova from 2005 to 2010.

**Figure 10:**
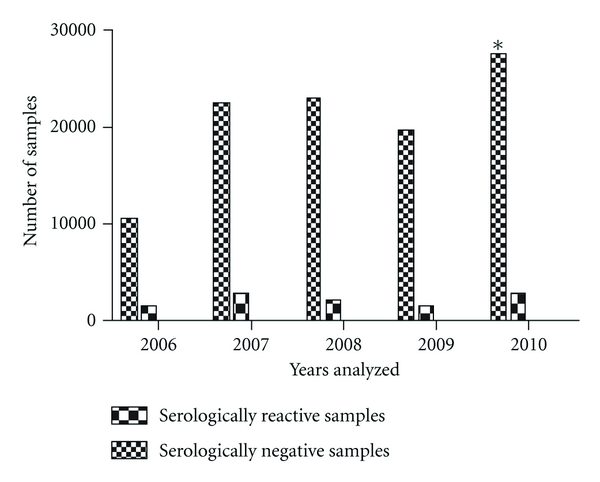
Chi-square testes to compare the proportions of serologically positive samples from 2006 to 2010. (There is no significant difference between 2007 and 2008 (*P* : 0.0874), the comparisons between the others pairs of years are significant different (*P* < 0.0001). The proportion in 2010 is significantly greater than in 2009 (*P* < 0.0014)—*Z* test for proportions).

The Plan for Intensification guaranteed the hiring of agents to control endemics diseases, purchase of personal protection equipment, laboratory equipment, sprayers, reform of the physical area, and increasing processing capability of the Laboratory for Zoonoses to 18,000 samples per month, the hiring of an expert to develop a specific information system for the program of leishmaniases, production of information materials, staff training, among others [13]. Venda Nova SD could expand the capacity of organization and production of control activities to VL, encompassing aspects relating to technical and operational structures. This situation becomes clear when we analyze the significant increase in the number of samples collected in serosurveys and the tendency to regularize the number of buildings sprayed with insecticides (Figure 7). 

The MSH ranked the areas of disease transmission, based on the cumulative incidence of human cases in each CA. Other specific indicators of CA are added such as rates of seropositive dogs, environmental conditions favorable for the transmission and vulnerability index to health risk. This categorization is fundamental for the planning, selection, and definition of measures to be undertaken in the different CA particularly the canine serosurveys and vector control.

Venda Nova SD is improving the process of developing the programs to control VL adding specific information on the analysis. These analyses of previous years measures made possible a better assessment of actions and their results after the implementation of the SCZOO. These analyses have been improved in 2010 with the training technical professionals in MapInfo software.

Rates of positivity to canine exams from 2006 to 2010 show that the canine infection is an expansion process on SD. VL is an emerging disease in BH mainly concentrated in underprivileged areas, with a number of factors which contribute to its occurrence [14]. However, as occurred to VL human cases in SD Venda Nova is clearly noticed that the occurrence of serologically positive dogs is spread out throughout all geographical area of SD, and areas of concentration of serologically positive dogs occur both in risk areas and outside them (Figure 3).

Despite the tendency of stabilization or decline in human cases in SD, is evident a necessity for additional entomological and environmental indicators to support the control measures. Several scientific works on leishmaniases have been developed in BH, including those allowed characterize their sand flies fauna, there is no systematic measure that evaluated the effectiveness of control measures on the vector population.

Studies on the phlebotomine fauna in BH demonstrated the presence of vector of *Lesihmania* which are etiologic agents of VL and MCL [15, 16]. A study conducted from 2001 to 2003, covering all SD of BH City reported the presence of 15 species with the predominance of *Lutzomyia longipalpis*. Vector of *Leishmania braziliensis* as *Nyssomyia whitmani* and *Nyssomyia intermedia* has also been reported [16]. Other study conducted in Northeast SD from 2006 to 2007 records important vector species and high natural infection rates which indicate the strength of infection to *Leishmania infatum* in Northeast SD. Natural infection rates for *Leishmania infatum* were 14.3%, 3.80%, 19.0% for *Nyssomyia whitimani, Nyssomyia intermedia,* and *Lutzomyia longipalpis,* respectively [17].

Environmental surveillance, considering the use of vector attributes, should be focused on ecological, epidemiological, climatic, and social factors engaged in domestication and urbanization of insects [18]. However, even though the adaptability of sand fly vectors to anthropic-modified environments could be one of the factors related to the increase of leishmaniases in BH, others no less important are related to new habits of population in occupation of the urban space, which eventually provide contact among human beings, vectors, and reservoirs [19–21].

Regarding this perspective, the environmental management strategy that is being implemented by SD Venda Nova could favor the control VL and other urban diseases. Several studies point out the need to modify the environment of the backyards of houses to control urban health problems. Another paper pointed that the use of spatial analysis tools for epidemiological surveillances indicate the need to correlate the vector density with peridomestic environmental aspects [22].

SMH worked intensively to control LV. A total of 752,243 samples of canine blood were analyzed, 50451 seropositive dogs were euthanized, and 496,397 buildings were sprayed from 2006 to 2010 [9]. These data suggest that only the removal of seropositive dogs and spraying of buildings does not control the occurrence of infection in dogs by *Leishmania* sp. One example of this was described in the municipality of Araçatuba where 45% houses with a dog euthanized due to leishmaniases replaced the dog in a period of one year [23].

A change of view is necessary on the occurrence of this disease in urban areas where the disease has characteristics yet unknown. Analyses of data distributed in the geographic spaces are increasingly appreciated in health management, it points useful information for the planning and evaluation of actions, based on the distribution of disease, location of health services, and environmental characteristics [24]. An example can be observed in the change of the programming from 2006 to 2010 and the programming to 2011 in SD Venda Nova, the access to information and the possibility to mapping the events can change the control strategies.
